# The Microtubule Severing Protein Katanin Regulates Proliferation of Neuronal Progenitors in Embryonic and Adult Neurogenesis

**DOI:** 10.1038/s41598-019-52367-3

**Published:** 2019-11-04

**Authors:** Franco L. Lombino, Mary Muhia, Jeffrey Lopez-Rojas, Monika S. Brill, Edda Thies, Laura Ruschkies, David Lutz, Melanie Richter, Torben J. Hausrat, André T. Lopes, Francis J. McNally, Irm Hermans-Borgmeyer, Jessica E. M. Dunleavy, Sabine Hoffmeister-Ullerich, Michael Frotscher, Thomas Misgeld, Michael R. Kreutz, Froylan Calderon de Anda, Matthias Kneussel

**Affiliations:** 1 Department of Molecular Neurogenetics, ZMNH, University Medical Center Hamburg-Eppendorf, Falkenried 94, 20251 Hamburg, Germany; 2 RG Neuroplasticity, Leibniz Institute for Neurobiology, 39118 Magdeburg, Germany; 3 Institute of Neuronal Cell Biology, Technical University Munich, Biedersteiner Straße 29, 80802 Munich, Germany; 4 Department of Structural Neurobiology, ZMNH, University Medical Center Hamburg-Eppendorf, Falkenried 94, 20251 Hamburg, Germany; 5 Research Group of Neuronal Development, ZMNH, University Medical Center Hamburg-Eppendorf, Falkenried 94, 20251 Hamburg, Germany; 6 Department of Molecular and Cellular Biology, University of California Davis, 95616 Davis, CA USA; 7 Transgenic Facility, ZMNH, University Medical Center Hamburg-Eppendorf, Falkenried 94, 20251 Hamburg, Germany; 8 Male Infertility and Germ Cell Biology Lab, School of Biological Sciences, Monash University, VIC, 3800 Australia; 9 Bioanalytics Facility, Center for Molecular Neurobiology, ZMNH, University Medical Center Hamburg-Eppendorf, Falkenried 94, 20251 Hamburg, Germany; 10 Leibniz Group Dendritic Organelles and Synaptic Function, ZMNH, University Medical Center Hamburg-Eppendorf, Falkenried 94, 20251 Hamburg, Germany

**Keywords:** Neural progenitors, Neuronal development

## Abstract

Microtubule severing regulates cytoskeletal rearrangement underlying various cellular functions. Katanin, a heterodimer, consisting of catalytic (p60) and regulatory (p80) subunits severs dynamic microtubules to modulate several stages of cell division. The role of p60 katanin in the mammalian brain with respect to embryonic and adult neurogenesis is poorly understood. Here, we generated a *Katna1* knockout mouse and found that consistent with a critical role of katanin in mitosis, constitutive homozygous *Katna1* depletion is lethal. Katanin p60 haploinsufficiency induced an accumulation of neuronal progenitors in the subventricular zone during corticogenesis, and impaired their proliferation in the adult hippocampus dentate gyrus (DG) subgranular zone. This did not compromise DG plasticity or spatial and contextual learning and memory tasks employed in our study, consistent with the interpretation that adult neurogenesis may be associated with selective forms of hippocampal-dependent cognitive processes. Our data identify a critical role for the microtubule-severing protein katanin p60 in regulating neuronal progenitor proliferation *in vivo* during embryonic development and adult neurogenesis.

## Introduction

During corticogenesis, excitatory neurons of the mammalian neocortex originate from proliferative radial glial cells (RGCs) extending fibers to the pial surface^1^. In rodents, RGCs are restricted to the ventricular zone (VZ) and undergo multiple rounds of asymmetric divisions to produce intermediate progenitor cells (IPCs)^2,3^. In contrast to RGCs, IPCs lack apical-basal polarity^4^. They occupy the subventricular zone (SVZ) and divide to generate neurons that migrate along RGC fibers towards the cortical plate (CP)^5^. IPCs undergo only one round of division to produce two neurons^6^. Several factors have been shown to regulate neural stem cell division and differentiation during cortical development^1^. For instance, triple deletion of APP and its paralogues APLP1 and APLP2 causes cortical progenitors to remain in their undifferentiated state much longer. This condition increases mitotic cells but does not impact migration^7^.

The adult hippocampus harbors neural stem cells in the subgranular zone (SGZ) of the dentate gyrus (DG). Upon activation, they self-renew and generate intermediate neuronal progenitors (NPCs) that subsequently differentiate into neuroblasts, dentate granule cells (DGCs) and astrocytes^8,9^. These processes, which include proliferation, differentiation, migration, and integration, are mediated by different signaling molecules, transcription factors, cytoskeletal elements, and membrane proteins^10^.

The organization and dynamics of microtubules (MTs) are fundamental for cell division and migration^11^. MT dynamics rely on a fine balance between polymerization and depolymerization at their plus- and minus-ends, including lattice breaks through severing^12^. The AAA-ATPase MT severing proteins, which include katanin, spastin, and fidgetin, hydrolyze ATP to catalyze severing of long MTs into short forms^13–16^. Katanin regulates spindle formation underlying meiotic and mitotic cell divisions^17–19^. Katanin is a heterodimer consisting of 60 kDa catalytic and 80 kDa regulatory subunits, but also contains additional katanin p60-like proteins^14^. The complex severs MTs also from their ends^20^ and binds to ASPM and CAMSAP, which regulate its MT minus-end severing activity^21,22^.

In neurons, katanin releases MTs from the centrosome^23^ and regulates axonal growth^24,25^. The MT-associated protein tau protects MTs from katanin severing^26^ and MT acetylation regulates the sensitivity to MT severing by katanin^27^. Inactivation or mutation of katanin p80 revealed the role of katanin-mediated MT-severing in male gamete production and the regulation of centriole and cilia number during brain development^28,29^. Presently, the role of katanin p60 *in vivo* as regards embryonic cortical neurogenesis remains unclear. Moreover, little is known on whether katanin p60 contributes to adult hippocampal neurogenesis, plasticity and hippocampal-dependent cognitive functions. Here, we generated a *Katna1* knockout mouse model to investigate the contribution of katanin p60 in the embryonic and adult brain. Our study identifies an important function for the microtubule-severing protein p60 katanin in embryonic survival, and highlights its role in neuronal progenitor proliferation in the developing cortex and during adult hippocampal neurogenesis.

## Results

### Katanin p60 is essential for embryonic survival

**Figure 1:**
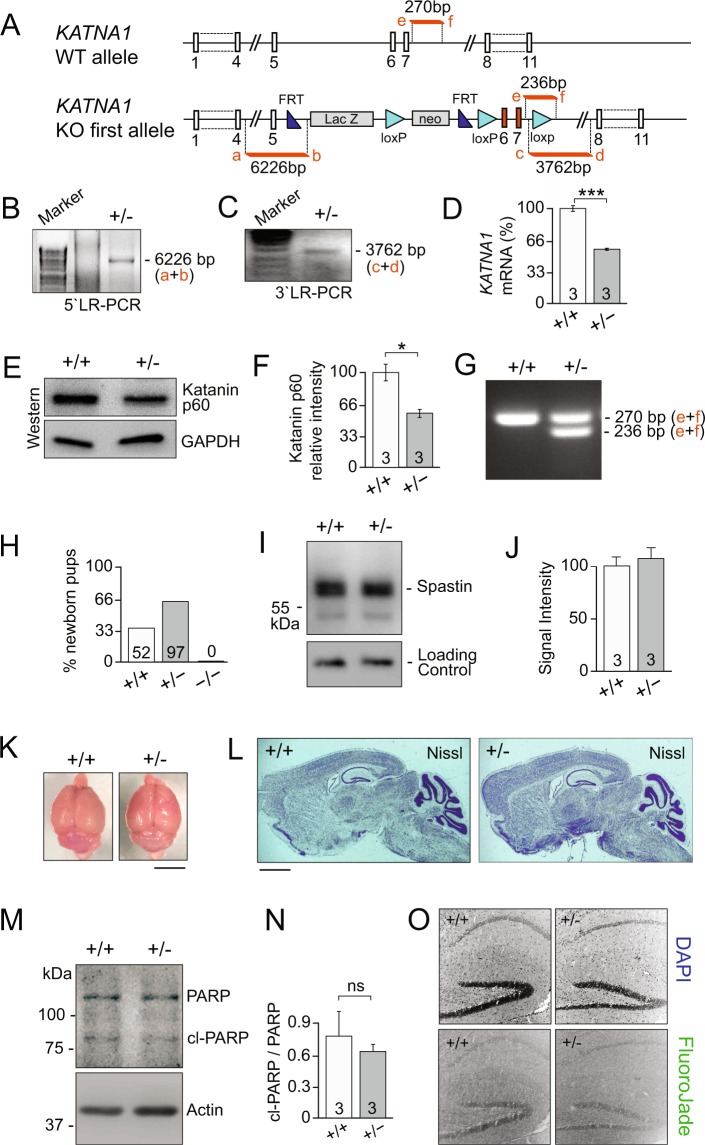
Homozygous depletion of p60 katanin leads to embryonic lethality. (**A**) Scheme of *Katna1* gene targeting strategy. (**B**) Long-range PCR over 5′ targeting vector integration site. (**C**) Long range PCR over 3′ targeting vector integration site. (**D**) Quantification of mRNA levels through RT-PCR and q-PCR, n = 3 ***p < 0.001. (**E,F**) As expected based on the genetic approach and observed mRNA levels, quantification of katanin p60 protein expression levels through western blotting, n = 3. Data represented as mean ± S.E.M., *p < 0.05. Independent samples T-test (One-tailed). (**G**) Genotyping PCR, WT band 270 bp, KO-first band 236 bp. (**H**) Number of newborn pups per genotype. (**I,J**) Quantification of spastin expression levels in adult mice by western blotting, n = 3. (**K**) Representative images show comparable sizes of adult brains dissected from heterozygous (+/−) mice and control (+/+) littermates. (**L**) Nissl staining showing overall normal gross brain anatomy.   (**M,N**) Quantification of of PARP cleavage to analyze apoptosis, n = 3. (**O**) Analysis of Fluoro-jade to label degenerating neurons. DAPI labeling detects all cells. Wild-type (+/+) littermates, heterozygous (+/−), and homozygous (−/−) katanin p60 knockout mice.

To investigate the role of p60 katanin *in vivo*, we generated *Katna1* knockout (−/−) mice using knockout first embryonic stem cells (KOMP, clone No. 44425) (Fig. 1A). Gene targeting was verified using long-range PCRs spanning the 5′ and 3′ genomic integration sites (Fig. 1B,C). RT-PCR (Fig. 1D) and western blotting using a p60-specific antibody^17^ (Fig. 1E,F) confirmed reduced katanin mRNA and protein expression in heterozygous (+/−) animals. PCR-Genotyping (Fig. 1G) revealed a mendelian 1:2 ratio of newborn +/+ and +/− pups. Notably, no viable homozygous (−/−) mice were obtained out of 149 animals born (Fig. 1H). We obtained a necrotic homozygous knockout (−/−) embryo at E15 but not at later stages, suggesting that complete p60 depletion results in prenatal lethality. Expression levels of katanin’s functional homolog spastin were unaltered at pre- and postnatal stages (Fig. 1I,J and data not shown). Initial characterization revealed that brain sizes and overall brain anatomy were unchanged in heterozygous (+/−) mice compared with control (+/+) littermates (Fig. 1K,L). Furthermore, there was no evidence of apoptosis or neuronal degeneration in heterozygotes using PARP-1 cleavage and fluorojade-C labeling, respectively (Fig. 1M–O). These findings indicate a critical role for p60 katanin in embryonic survival and are consistent with its central function in MT regulation during mitosis^18,30,31^.

### Katanin p60 haploinsufficiency affects cell positioning during corticogenesis

**Figure 2:**
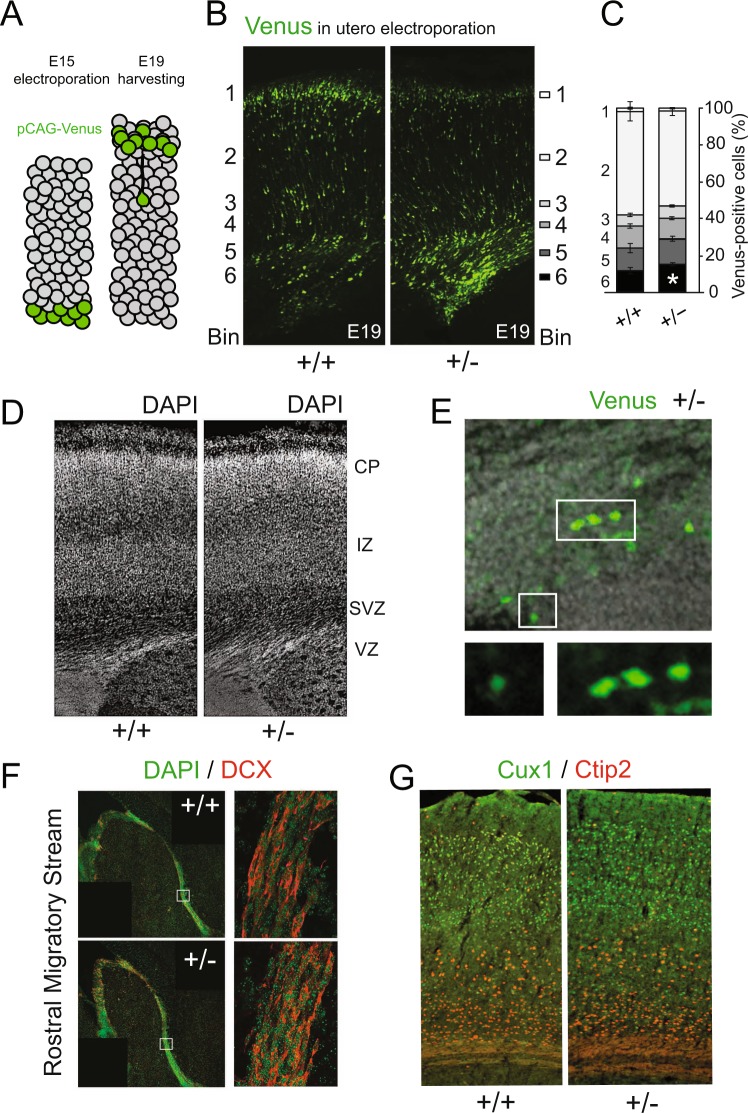
Katanin p60 regulates neuronal progenitor positioning during corticogenesis. (**A–C**) E15 *in utero* electroporation followed by E19 analysis revealed a significant increase of pCAG-Venus-positive cells at the ventricular/subventricular zone of the developing cortex. Non-parametric T-test (Two-tailed). (+/+) n = 4, 16 sections; (+/−) n = 7, 26 sections. For Bin 6 (+/+) = 11.53%  ± 1.81%; (+/−) = 15.64%±0.88%. Data represented as mean ± S.E.M. *p < 0.05. (**D**) DAPI staining at E19. (**E**) Accumulating cells display a rounded shape in contrast to the typical bipolar shape of migrating cells. (**F**) DCX-positive cells display normal RMS migration in adult animals. [Boxed regions are enlarged and show comparable positioning of DCX-positive cells between the two genotype groups]. (**G**) In contrast to stage E19, Cux1 and Ctip2 staining in sagittal sections of young adult mice show comparable layering between genotypes.

Katanin p60 is implicated in cell migration as it localizes to the cell cortex to modulate cell motility^20^. Moreover, it is highly expressed by cells in the neurogenic niche of the embryonic cortex^32^, suggesting that it may play an essential role in corticogenesis, during which the cortex develops in an inside-out manner^33^. To address this, we expressed fluorescent Venus using *in utero electroporation* at stage E15 and analyzed the position of labeled cells four days after (Fig. 2A). Quantification of Venus-positive cells across six cortical bins revealed that cells from heterozygotes in general arrived at the upper cortical layers (Fig. 2B,C). Cortical anatomy appeared normal in heterozygous (+/−) mice (Fig. 2D), although we cannot fully exclude that other cell types partly contributed to this effect. However we observed a significant accumulation of Venus-positive cells at the VZ and SVZ (Fig. 2A–C, Bin 6). Based on their lack of apical-basal polarity, we identified the accumulating cells as neuronal progenitors (Fig. 2E). To investigate whether neuronal migration in general was impaired by p60 deficiency irrespective of developmental stage, we analyzed doublecortin (DCX)-positive cells in the rostral migratory stream (RMS), a region along which adult neuronal precursors, originating from the SVZ, migrate to reach the main olfactory bulb. We observed similar cell distributions along the RMS in young adult mice (Fig. 2F). Further analysis of cortical layers revealed that the observed accumulation of cells in VZ/SVZ (compare with stage E19, Fig. 2A,B) did not lead to malformation or aberrant cell distribution of layers II, III, IV (Cux1) or layers V, VI (Ctip2) (Fig. 2G). These findings indicate that reduced p60 katanin gene expression, likely leading to reduced MT severing, primarily delays neuronal proliferation in the cortical neurogenic niche. However, this defect does not overtly affect the cortical architecture during embryonic development or at postnatal stages.

### Katanin p60 haploinsufficiency impairs the positioning of newborn neurons at the dentate gyrus GCL without affecting LTP or learning and memory

**Figure 3:**
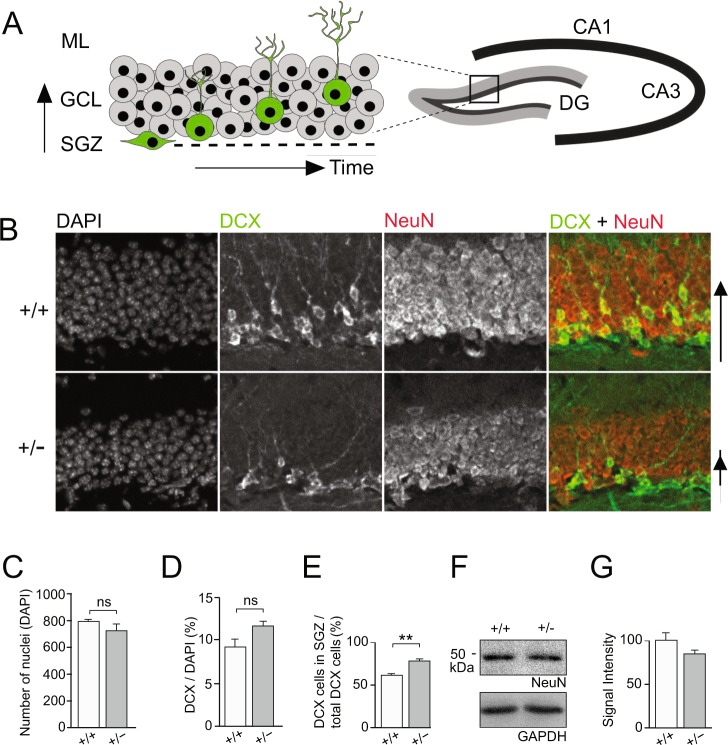
P60 katanin regulates newborn neuron positioning during neurogenesis in the adult hippocampus. (**A–D**) Immunohistochemical evaluation of DG shows no difference in the total number of DAPI−, Doublecortin (DCX)- or NeuN-positive cells, but reveals an accumulation of DCX+ neuronal progenitors in the subgranular zone (SGZ) of the DG, in p60 +/− mice. (+/+) n = 3; (+/−) n = 3. (+/+) = 61.4 ± 1.51; (+/−) = 76.4 ± 1.25. Data represented as mean percentage ± S.E.M. **p < 0.01 independent samples T-test (Two-tailed). (**E**,**F**) Equivalent expression levels of the post-mitotic marker NeuN in p60 +/+ and +/− hippocampal lysate.

To investigate whether katanin p60 haploinsufficiency also affects neuronal progenitors in adulthood, we focused our analysis on adult hippocampal neurogenesis. Doublecortin (DCX)-immunostaining was used to visualize IPCs and young neurons in the DG (Fig. 3A,B). The total cell numbers (Fig. 3B,C), and the ratio of immature DCX-positive cells to total cells (DAPI) (Fig. 3B,D) was comparable for both genotypes. Interestingly, the location of DCX-positive cells was significantly restricted to the SGZ in heterozygous (+/−) mice (Fig. 3B,E). This was despite comparable NeuN expression levels, indicative of mature neurons, between the two genotypes (Fig. 3B,F,G).

**Figure 4:**
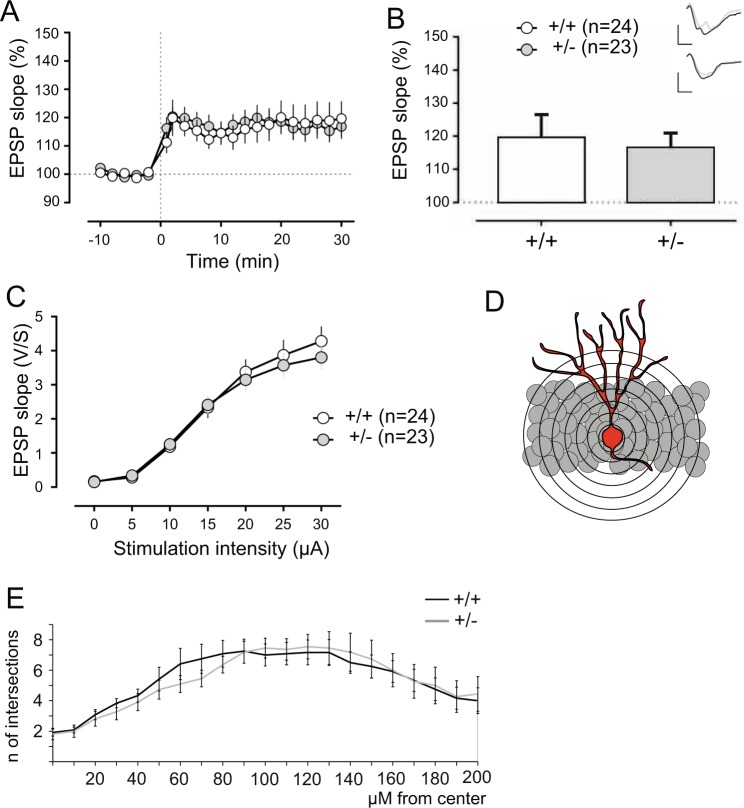
Katanin p60 haploinsufficiency does not affect long-term potentiation at the dentate gyrus. (**A**) Long-term potentiation (LTP) at the medial perforant pathway following a high-frequency stimulation protocol. (**B**) Both genotypes showed similar synaptic potentiation based on the mean EPSP slope during the last 5 min of recordings. Insets show analogue traces of EPSPs recorded during baseline and 30 min after high-frequency stimulation. Scale bars: 2 mV/2 ms. (**C**) There were no significant EPSP slope differences in basal conditions between genotypes. (**D,E**) Sholl analysis of dentate gyrus granule cells show no significant differences in dendritic arborization between genotypes.

Since adult neurogenesis is required for synaptic transmission and plasticity in the dentate gyrus^34,35^, we therefore asked whether the mislocalization of DCX-positive cells may impact DG synaptic plasticity in heterozygous (+/−) mice. To this end, we performed extracellular field recordings following high-frequency stimulation (HFS) of the medial perforant pathway. Relative to baseline, we observed significant potentiation of DG evoked responses 30 min after HFS induction (Fig. 4A) as demonstrated by comparable fEPSP slopes in both genotype groups (Fig. 4B). Moreover, basal synaptic transmission was similar in slices derived from both genotype groups (Fig. 4C). This was consistent with normal DG granule cell morphology, as evidenced by comparable dendritic arborization in heterozygotes (+/−) and control (+/+) neurons (Fig. 4D,E). Therefore, although heterozygous katanin p60 knockout induces a mislocalization of DCX-positive cells in the DG (Fig. 3), it does not modify persistent strengthening of hippocampal DG synapses.

**Figure 5:**
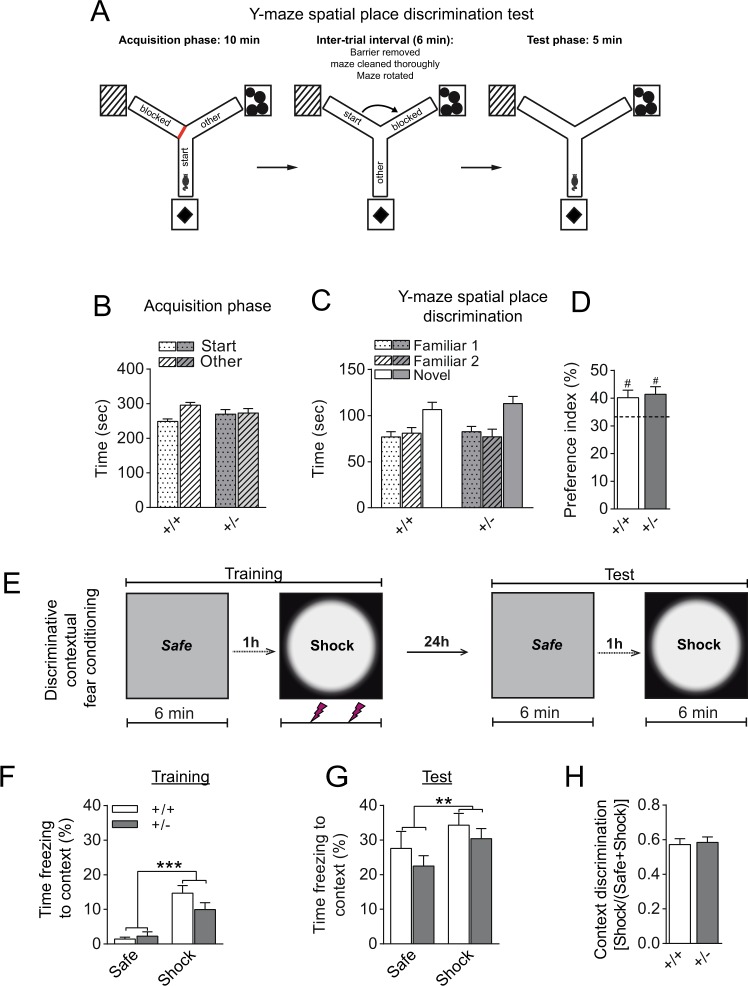
Katanin p60 haploinsufficiency does not alter hippocampal-dependent spatial and contextual memory. (**A**) Schematic representation of the spatial Y-maze procedure used to examine the capacity to employ allocentric strategies to explore a previously inaccessible location. (**B**) Equivalent time spent in both arms during the acquisition phase indicates that motivation to explore is unaltered in heterozygous (+/−) mice. (**C,D**) In the test phase, both genotype groups showed biased exploration of the previously unfamiliar spatial location. ^#^p < 0.05 based on a one-sample t-test against chance level (dashed line) preference (33.3%) for the novel location. (**E**) Scheme of experiment protocol used to examine discrimination between two distinct contexts in a fear conditioning paradigm. (**F**) Both genotype groups showed significantly more freezing responses to the shock-paired context compared with the safe context (Context: ***p < 0.0001), +/− mice showed delayed capacity to form context-shock association (Context × Genotype: p = 0.038) during the training phase. (**G**) A 24-h retention test revealed significantly higher freezing responses to the shock context compared with the safe context for both genotype groups (Context: **p < 0.01). (**H**) Comparable context discrimination index between the two genotype groups indicated intact context discrimination and memory consolidation in heterozygous (+/−) mice. Values represent mean ± SEM.

In keeping with this, katanin p60 haploinsufficiency did not alter learning and memory processes shown to rely on adult hippocampal neurogenesis^36–40^. Initial assessment of general behavioral function revealed intact anxiety-related behavior, locomotor activity and spontaneous alternation in heterozygous (+/−) mice (Supplementary Figure S1A–F). Further evaluation in the hippocampal-dependent Y-maze spatial novelty preference test (Fig. 5A–D) and context discrimination in fear conditioning (Fig. 5E–H) revealed comparable performance between heterozygous (+/−) mice and control (+/+) littermates.

Taken together, these findings indicate that katanin p60 haploinsufficiency retains neuronal precursors and new-born post-mitotic neurons in the dentate gyrus (DG) SGZ (Fig. 3). Yet, these deficits are insufficient to perturb long-term potentiation (LTP) in DG or hippocampal-dependent spatial and contextual learning and memory (Figs 4,5).

### Katanin p60 regulates neuronal precursor proliferation in the adult DG

**Figure 6:**
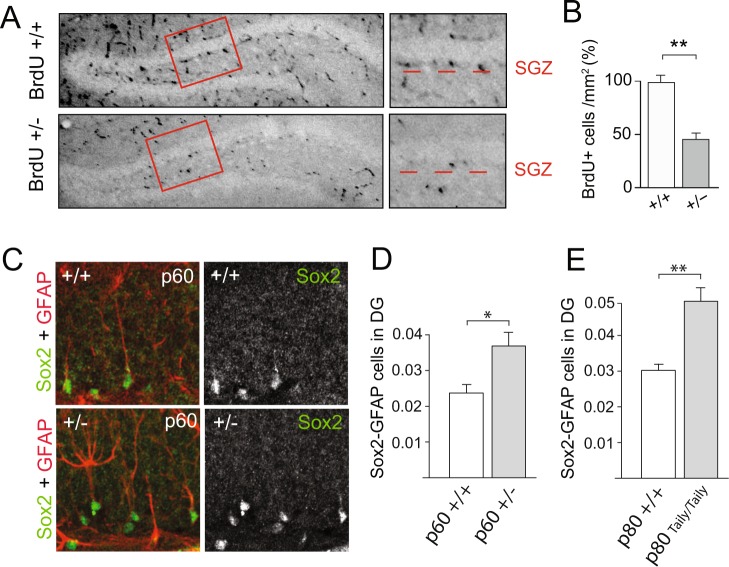
Katanin p60 regulates adult dentate gyrus neurogenesis. (**A,B**) BrdU-positive cells in the SGZ are significantly reduced in the dentate gyrus of p60 heterozygous (+/−) animals. (+/+) n = 28 hippocampi, 3 animals; (+/−) n = 28 hippocampi, 3 animals. (+/+) = 99.663% ± 6.927%; (+/−) = 44.530% ± 6.038%. Independent samples T-test (Two-tailed). Data represented as mean ± S.E.M. ***p < 0.0001. (**C,D**) Sox2/GFAP co-immunostaining reveals expansion of immature neuronal progenitor cells upon katanin p60 downregulation. (+/+) n = 4; (+/−) n = 3. (+/+) = 0.024 ± 0.002; (+/−) = 0.037 ± 0.004. Independent samples T-test (Two-tailed). Data represented as mean ± S.E.M. *p < 0.05. (**E**) Sox2/GFAP co-immunostaining reveals a similar expansion of immature neuronal progenitor cells upon depletion of the regulatory katanin p80 subunit. (+/+) n = 3; (Taily/Taily) n = 4. (+/+) = 0.029 ± 0.002; (Taily/Taily) = 0.051 ± 0.006. Mann-Whitney U Test (Two-tailed). Data represented as mean ± S.E.M. **p < 0.01.

Mislocalization of DCX+ cells can arise from either impaired proliferation of precursor cells or altered migration of young post-mitotic neurons. Since *Katna1*+/− neurons in general reach the cortical plate and migrate along the RMS (Fig. 2), we aimed to understand whether p60 haploinsufficiency primarily affects the division of neuronal precursors in the SGZ. First, we examined the incorporation of the synthetic nucleoside 5-bromo-2′-deoxyuridine (BrdU) into newly synthesized DNA of replicating cells. Quantification of BrdU-positive cells in the dentate gyrus SGZ revealed a significant reduction in the number of proliferating cells of to about 50% in *Katna1*+/− neurons (Fig. 6A,B). This indicates that reduced p60 katanin expression levels affect the cell cycle of proliferating cells in the adult brain, consistent with a critical role for p60 katanin and microtubule severing in cell division^17–19^. Second, we performed Sox2/GFAP double-immunostaining to label immature type 1 neuronal precursor cells^41^ (Fig. 6C). Quantification of double-positive cells in the SGZ revealed a significant increase in immature type-1 cells in *Katna1*+/− mice (Fig. 6D). As the generation of type-2 IPCs requires asymmetric cell division of type-1 RGCs^41^, these data are consistent with our results from BrdU labeling and support the view that p60 katanin is required for the proliferation of neuronal progenitors. To confirm this interpretation, we tested mutant mice containing a *Katnb1* loss-of-function allele (Taily mutation), which in wildtype mice encodes katanin’s regulatory p80 subunit^29^. Likewise, and consistent with our findings, Sox2/GFAP staining in the DG of katanin p80-deficient mice yielded comparable results (Fig. 6E), thus confirming the critical role of the entire katanin severing complex in DG cell division.

Altogether, our study highlights a role for katanin p60 in regulating the proliferation of neuronal progenitors *in vivo* during embryonic corticogenesis and adult hippocampal neurogenesis.

## Discussion

Dynamic MTs are fundamental for different aspects of cell division, including centrosomal positioning, spindle formation/function and cytokinesis^11^. Here, we generated a constitutive *Katna1* knockout mouse to investigate the role of p60 katanin in embryonic and adult neurogenesis. Due to the central role of the katanin complex in severing MTs^23^, homozygous katanin p60 depletion results in embryonic lethality. Katanin p80 contributes to neuronal motility in the mouse developing cortex, and mutations in *Katnb1* lead to microlissencephaly in humans^28^. Homozygous *Katnb1* mutant mice are embryonically lethal, possibly because of impaired erythropoiesis^28^. Alternatively, it was shown that p80 depletion affects left-right asymmetry leading to embryonic malformations and cardiac deficits, which reflects an impaired MT network and cilia^42^. Our study indicates that the catalytic p60 subunit, which is a component of the katanin severing complex, plays an equally important role for embryonic survival. This outcome, including specific deficits during corticogenesis and adult hippocampal neurogenesis, possibly stemmed from impaired cell proliferation (Fig. 6).

Katanin p60 is implicated in cell migration as previous studies have shown that it localizes to the cell cortex to modulate cell motility^20^. Others have shown that katanin p60 is highly expressed by cells in the neurogenic niche of the embryonic cortex ventricular zone and participates in neuronal positioning via interactions with NDEL1 and Lis 1^32^. Consistent with its expression profile, our study highlights a role for katanin p60 during corticogenesis by demonstrating that p60 haploinsufficiency impairs cell proliferation at the neurogenic niche. Although embryonic and adult neuronal migration undergo different mechanisms, our assessment of neurons at the embryonic cortical plate, including long distance migration in the adult RMS (Fig. 2), suggests that general neuronal migration occurs irrespective of the developmental stage. We cannot fully exclude that neuronal migration was completely unaffected, but our data support the interpretation that katanin p60 haploinsufficiency primarily impairs neuronal precursor proliferation. Consequently, neuronal progenitors may be retained in their undifferentiated state much longer, since such an outcome has previously been shown to increase the number of mitotic cells without severely affecting neuronal migration^7^. Indeed, this may delay the rate of neuronal migration, but ultimately neurons arrive at their respective destinations. Along these lines, our observations of normal cortical layering in the adult cortex (Fig. 2), implies that katanin p60 haploinsufficiency may delay neurogenesis during embryonic cortical development. This effect might have been restored at more mature stages by compensatory effects of spastin severing activity or the two p60 katanin-like subunits (katanin p60-like1 and p60-like2)^43,44^. Alternatively, katanin p60 function during corticogenesis may be restricted to a specific time window.

The adult mammalian brain generates newborn neurons at two neurogenic areas: the subventricular zone (SVZ) and the subgranular zone (SGZ) of the DG^45,46^. In the SGZ, astroglial progenitors and neuronal precursor cells proliferate and produce intermediate precursor cells that ultimately proliferate and generate new neurons that can be distinguished by specific markers^41^. A widely used marker is the MT-associated protein DCX, which is highly expressed in late stages of IPCs and young-migrating neurons^41^. In this study, we observed a significant accumulation of DCX-positive cells in the dentate gyrus SGZ of adult p60 katanin heterozygous knockouts (Fig. 3). These results resembled our findings during embryonic corticogenesis were cells accumulated in the neurogenic niche (Fig. 2), indicating that katanin p60 plays a critical role in regulating neuronal progenitor proliferation during embryonic development as well as adult neurogenesis. Labeling of proliferating cells with BrdU showed a significant reduction of cells that entered the cell cycle (Fig. 6). In combination, Sox2 and GFAP label astroglial progenitors, NPCs and early stages of IPCs^41^. These progenitors were significantly increased in p60 katanin heterozygous knockouts (Fig. 6), suggesting for delays or impairments in the cell cycle of this progenitor population. Interestingly, these results were phenocopied in a mouse mutant characterized by loss-of-function of the regulatory p80 subunit (Fig. 6E), thereby confirming that the katanin complex indeed plays a critical role in proliferation of progenitors. As such, a stoichiometric balance between regulatory and catalytic katanin subunits is relevant to this process.

Despite altering neuronal progenitor proliferation, our study shows that p60 heterozygous knockout does not negatively impact long-term potentiation (LTP) at the adult hippocampus DG (Fig. 4). These results are in contrast to a previous study, which applied gamma radiation to the brain to reduce cell proliferation^35^. However, to interpret these discrepancies, it needs to be investigated to which extent gamma radiation doses unspecifically affect neuronal cell death. Furthermore, deficits in long-term potentiation have been shown to recover within several weeks following the ablation of neurogenesis^40^, a phenomenon that may include synaptic rewiring.

Former studies on the relationship of adult neurogenesis and hippocampal-dependent behavior came to controversial conclusions to which extent adult newborn neurons participate to cognitive performance^36–39^. It should be noted that a decrease in adult neurogenesis does not necessarily mean that the adult DG would no longer be provided with new neurons. In fact, a significant percentage of adult-generated neurons anyway undergo selective apoptosis, while only the remaining cells integrate into the DG circuitry^47^. It is therefore plausible to conclude that a reduction of DG neurogenesis in the range of 50% in *Katna1* heterozygotes remains sufficient to provide the DG circuitry with newborn neurons.

In support of this view, our Sholl analysis of adult DG granule neurons, revealed an equal dendritic arborization among genotypes, suggesting that despite neurogenesis was decreased, the pool of generated neurons that resisted selective apoptosis reached maturation and was successfully integrated in the DG circuitry. This was sufficient to support learning and memory of hippocampal-dependent tasks in p60 heterozygous (+/−) knockout mice.

In summary our data highlight a critical role of p60 katanin in embryonic survival and the proliferation of neuronal progenitors at both embryonic and young adult stages.

## Materials and Methods

Additional materials and methods are provided in the Supplementary Information.

### Generation of knockout mice

The generation of katanin knockout mice and *in vivo* experiments were conducted in accordance with the German and European Union laws on protection of experimental animals following approval by the local authorities of the City of Hamburg (Committee for Lebensmittelsicherheit und Veterinärwesen, Authority of Soziales, Familie, Gesundheit und Verbraucherschutz Hamburg, Germany, No. 90/10 and 100/13). The targeting vector (KOMP, clone No. 44425) was generated by the trans-NIH knock-out project and obtained from the KOMP repository (www.komp.org). Gene targeting in JM8A3 ES cells, obtained from the European Conditional Mouse Mutagenesis consortium (EUCOMM; Pettitt *et al*., 2009; Skarnes *et al*., 2011), was performed according to the protocol provided by the International Knockout Mouse Consortium (IKMC). Positive clones were identified by Southern blot analysis. Chimeric males were obtained by injection of the ES cells into C57BL6/J 8-cell stage embryos and were mated with C57BL6/J WT female mice to establish germline transmitted founders. Offspring were crossed to produce homozygous offspring. Heterozygous ‘knockout-first’ offspring with a minimum of N3 on a C57BL6/J background were intercrossed to obtain wild-type (WT) and homozygous KO animals.

### Antibodies and reagents

Katanin p60^17^; Spastin (Abcam, Sp6C6, ab77144); PARP (Cell signaling, 9542); NeuN (Millipore, MAB377); GAPDH (GeneTex, 6C5, Gtx28245); Cux1 (Santa Cruz, sc-13024); Ctip2 (Abcam, ab18465); BrdU (Bio-Rad, OBT0030S); GFAP (Sigma, G3893); DCX (Santa Cruz, C-18, sc8066; Millipore, ab2253); Sox2 (Abcam, ab97959; Millipore, ab5603); β-Actin (Sigma, AC15, A5441); FluoroJade-C (Millipore, AG325–30mg).

### In utero-electroporation

Fifteen days after vaginal plugs, pregnant female mice were given a pre-operative dose of buprenorphine (0.05−0.01 mg/kg body weight) by subcutaneous injections at least 30 min before surgery. Animals were then anesthetized using 2.5% isoflurane / O_2_ inhalation. Oxygen was delivered with a flow rate of 0.65 L / min and isoflurane was applied via a vaporizer (Föhr Medical Instruments). The uterine horns were exposed and Venus-expressing plasmid was microinjected into the lateral ventricles of the embryos. Five current pulses (50 ms pulse, 950 ms interval; 35 mV) were delivered across the heads of the embryos. Post-surgery, 2−3 drops of meloxicam (0.1−0.5 mg/kg body weight) were given orally through soft food for 96 hours. Four days after electroporation, animals were euthanized and brains were harvested and fixed in 4% PFA/PBS for 24 hours. Brains were then cryoprotected in 30% sucrose/PBS for 48 hours and frozen using Tissue Tek. 35 μm free floating coronal sections were collected sequentially. Sections were then stained with DAPI (1:1,000) for 1 hour at room temperature and mounted with Aqua Poly Mount 1 (Polysciences). Low magnification images were acquired with an Olympus laser scanning confocal microscope (TMFV1000, Olympus, Hamburg, Germany). Due to variability in Venus expression among slices, laser power was adjusted in order to capture the highest number of cells possible while avoiding saturation. Since cell numbers but not fluorescence intensity was assessed, the brightness/contrast was adjusted for images whenever applicable. With exception of one embryo, four subsequent slices per brain which belonged to similar regions were quantified. Cortical binning for quantification was based on nuclear density and was performed using ImageJ (NIH, Bethesda, Maryland, USA). Data were subjected to non-parametric analysis as detailed in the figure legends using SPSS (IBM Corp. Version 22.0).

### BrdU-DAB stainings

2 months old male mice were injected with 1.5 mg of BrdU (Sigma B5002−1G) following incubation for 3 hours. Thereafter, mice were deeply anesthetized with a ketamine-xylazine mix in 0.9% NaCl and perfused transcardially with 4% PFA in PBS (pH 7.4). Brains were harvested and post-fixed in the same fixative for 4−6 hours, cyroprotected in 30% Sucrose / PBS for 48 hours and frozen using TissueTek. 35 μm coronal non-adjacent free-floating sections were cut using a cryostat and collected every 5^th^ from the previous one. Before staining, DNA was denatured by incubating slices for 1 hour at 37 °C in the presence of 2 M HCl, washed 3 times for 5 min in PBS and Blocked for 1 hour in TBS, 0,5% Triton X−100, 5% BSA. Slices were incubated over-night in primary antibody (TBS, 3% BSA, 0,1% Triton X−100). The day after, sections were washed twice in PBS and DAB staining was performed following Vectastain Universal Elite ABC kit protocol (cat. No. PK−6200).

### Behavioral experiments

Experiments were carried out in 2 cohorts of male and female mice aged 8−13 weeks old. Mice in cohort 1 were tested in the elevated plus-maze, light-dark transition test, open field test and spatial place recognition. Mice in cohort 2 were tested for Y-maze spontaneous alternation and discriminative contextual fear conditioning.

#### Y-maze spatial place discrimination and novelty preference test

Spatial recognition memory was evaluated in a symmetrical Y-maze composed of three identical arms (Length, 50 cm; Width, 9 cm, spaced at 120°) enclosed by 14 cm high transparent walls (thickness 1 cm). The maze was placed on a rotating platform and elevated approximately 90 cm above the ground. It was located in a dimly lit room experimental room (50 lux in the center of the maze) containing a variety of extra-maze cues. Three prominent and distinct cues were also placed adjacent to each arm in order to facilitate arm-cue association. In the acquisition phase of the task, mice were exposed for 10 min to two arms of the maze (start and other arm) with access to the third arm blocked by a white plastic door. The inaccessible arm constituted the novel spatial location that was used during the test phase. The allocation of arms to a specific spatial location was counterbalanced for each experimental group. Thereafter, the subject was removed from the maze and placed in a holding cage for 6 min. During this time, the barrier to the inaccessible arm was removed, the maze rotated (120°), and thoroughly cleaned to minimize the use of olfactory cues inside the maze. Mice were re-introduced to an arm located in the spatial location that had been used in the acquisition phase and allowed to explore all three arms for 5 min (see Fig. 4A for the a schematic representation of this procedure). The proportion of time spent in each arm for both training phases was analyzed. In addition, time spent in the arm representing the novel spatial location was calculated as follows: (time spent in the novel arm / time spent in all arms) × 100% as an index for novelty preference.

### Discriminative contextual fear conditioning

The testing apparatus consisted of two NIR video fear conditioning systems (Med associates Inc., VT, USA) that were modified to create two contexts with distinct visual, olfactory and tactile features. One context comprised a fear conditioning chamber with a patterned back wall, solid white floor (except when used as a shock context, in which case the grid floor was used), and saturated with a lemon scent. The other chamber contained a curved white insert, solid white floor (except when used as a shock context, in which case the grid floor was used) and saturated with a vanilla scent. Training in both contexts was performed in a counterbalanced manner such that the safe context became the shock context for half the mice in each genotype. On day 1, mice were first introduced to the ‘safe’ context and allowed a 6 min exploration interval. One hour later, mice were introduced to the ‘shock’ context and allowed to explore for 2 min, after which they received 2 foot shocks (unconditioned stimuli: 1 s, 0.4 mA) separated by a 2 min inter-trial interval. This was followed by a 2 min stimulus free interval before the mice were removed from the chambers and returned to their home cages. The amount of freezing to the shock context on day 1 provided a measure of conditioned fear acquisition. Twenty-four hours later, mice were reintroduced to the safe context, followed by re-exposure to the shock context one hour later. Testing in each context was conducted for 6 min in the absence of the shock stimuli. The presence of conditioned fear responses or lack thereof upon testing in the neutral context was used to examine fear generalization. To determine that conditioned fear responses were specific to the training context, a context discrimination index based on percentage freezing levels was calculated as follows: Context discrimination = Shock/(Safe + Shock).

### Dentate gyrus long-term potentiation

Field recordings were performed in transversal hippocampal slices from 11 weeks old mice. The slices (400 μm thickness) were cut with a chopper in ice-cold ACSF solution and placed in an interface chamber at 32 °C. The ACSF solution contained in mM: 124 NaCl, 4.9 KCl, 2 MgSO_4_, 2 CaCl_2_, 1.2 KH_2_PO_4_, 25.6 NaHCO_3_ and 10 glucose, equilibrated with 95% O_2_/5% CO_2_. Slices were incubated for at least 2 hours before the start of the recordings. All experiments were done on slices maintained *in vitro* for 2–8 hours. The field-EPSP was measured with an electrode positioned at the middle of the molecular layer of the dentate gyrus. The medial perforant path was stimulated with biphasic constant current pulses (0.1 ms per half-wave duration) through a monopolar stimulation electrode. The high-frequency stimulation (HFS) protocol consisted of 4 trains of 100 pulses (0.2 ms per half-wave duration) at 100 Hz, inter-train interval 20 seconds.

## Supplementary information

Supplementary Info

## Data Availability

There are no restrictions on the availability of materials or information.

## References

[1] Dehay C, Kennedy H (2007). Cell-cycle control and cortical development. Nat Rev Neurosci.

[2] Miyata T (2004). Asymmetric production of surface-dividing and non-surface-dividing cortical progenitor cells. Development.

[3] Noctor SC, Flint AC, Weissman TA, Dammerman RS, Kriegstein AR (2001). Neurons derived from radial glial cells establish radial units in neocortex. Nature.

[4] Haubensak W, Attardo A, Denk W, Huttner WB (2004). Neurons arise in the basal neuroepithelium of the early mammalian telencephalon: a major site of neurogenesis. Proc Natl Acad Sci USA.

[5] Noctor SC, Martinez-Cerdeno V, Ivic L, Kriegstein AR (2004). Cortical neurons arise in symmetric and asymmetric division zones and migrate through specific phases. Nat Neurosci.

[6] LaMonica BE, Lui JH, Wang X, Kriegstein AR (2012). OSVZ progenitors in the human cortex: an updated perspective on neurodevelopmental disease. Curr Opin Neurobiol.

[7] Shariati SA (2013). APLP2 regulates neuronal stem cell differentiation during cortical development. J Cell Sci.

[8] Toda, T., Parylak, S. L., Linker, S. B. & Gage, F. H. The role of adult hippocampal neurogenesis in brain health and disease. *Molecular psychiatry*, 10.1038/s41380-018-0036-2 (2018). 10.1038/s41380-018-0036-2 PMC6195869 29679070

[9] Kempermann G, Song H, Gage FH (2015). Neurogenesis in the Adult Hippocampus. Cold Spring Harb Perspect Biol.

[10] Lledo PM, Alonso M, Grubb MS (2006). Adult neurogenesis and functional plasticity in neuronal circuits. Nat Rev Neurosci.

[11] Akhmanova A, Steinmetz MO (2015). Control of microtubule organization and dynamics: two ends in the limelight. Nat Rev Mol Cell Biol.

[12] Ghosh DK, Dasgupta D, Guha A (2012). Models, Regulations, and Functions of Microtubule Severing by Katanin. ISRN Mol Biol.

[13] Sharp DJ, Ross JL (2012). Microtubule-severing enzymes at the cutting edge. J Cell Sci.

[14] Roll-Mecak A, McNally FJ (2010). Microtubule-severing enzymes. Curr Opin Cell Biol.

[15] Kahn OI, Baas PW (2016). Microtubules and Growth Cones: Motors Drive the Turn. Trends Neurosci.

[16] Vale RD (1991). Severing of stable microtubules by a mitotically activated protein in Xenopus egg extracts. Cell.

[17] McNally FJ, Thomas S (1998). Katanin is responsible for the M-phase microtubule-severing activity in Xenopus eggs. Mol Biol Cell.

[18] McNally K, Audhya A, Oegema K, McNally FJ (2006). Katanin controls mitotic and meiotic spindle length. J Cell Biol.

[19] Mains PE, Kemphues KJ, Sprunger SA, Sulston IA, Wood WB (1990). Mutations affecting the meiotic and mitotic divisions of the early Caenorhabditis elegans embryo. Genetics.

[20] Zhang D (2011). Drosophila katanin is a microtubule depolymerase that regulates cortical-microtubule plus-end interactions and cell migration. Nat Cell Biol.

[21] Jiang K (2017). Microtubule minus-end regulation at spindle poles by an ASPM-katanin complex. Nat Cell Biol.

[22] Jiang K (2018). Structural Basis of Formation of the Microtubule Minus-End-Regulating CAMSAP-Katanin Complex. Structure.

[23] Ahmad FJ, Yu W, McNally FJ, Baas PW (1999). An essential role for katanin in severing microtubules in the neuron. J Cell Biol.

[24] Yu W (2005). Regulation of microtubule severing by katanin subunits during neuronal development. J Neurosci.

[25] Karabay A, Yu W, Solowska JM, Baird DH, Baas PW (2004). Axonal growth is sensitive to the levels of katanin, a protein that severs microtubules. J Neurosci.

[26] Qiang L, Yu W, Andreadis A, Luo M, Baas PW (2006). Tau protects microtubules in the axon from severing by katanin. J Neurosci.

[27] Sudo H, Baas PW (2010). Acetylation of microtubules influences their sensitivity to severing by katanin in neurons and fibroblasts. J Neurosci.

[28] Hu WF (2014). Katanin p80 regulates human cortical development by limiting centriole and cilia number. Neuron.

[29] O’Donnell L (2012). An essential role for katanin p80 and microtubule severing in male gamete production. PLoS genetics.

[30] Matsuo M (2013). Katanin p60 contributes to microtubule instability around the midbody and facilitates cytokinesis in rat cells. PLoS One.

[31] Joly N, Martino L, Gigant E, Dumont J, Pintard L (2016). Microtubule-severing activity of the AAA+ ATPase Katanin is essential for female meiotic spindle assembly. Development.

[32] Toyo-Oka K (2005). Recruitment of katanin p60 by phosphorylated NDEL1, an LIS1 interacting protein, is essential for mitotic cell division and neuronal migration. Hum Mol Genet.

[33] Paridaen JT, Huttner WB (2014). Neurogenesis during development of the vertebrate central nervous system. EMBO Rep.

[34] Massa F (2011). Conditional reduction of adult neurogenesis impairs bidirectional hippocampal synaptic plasticity. Proc Natl Acad Sci USA.

[35] Snyder JS, Kee N, Wojtowicz JM (2001). Effects of adult neurogenesis on synaptic plasticity in the rat dentate gyrus. J Neurophysiol.

[36] Shors TJ (2001). Neurogenesis in the adult is involved in the formation of trace memories. Nature.

[37] Dupret D (2008). Spatial relational memory requires hippocampal adult neurogenesis. PLoS One.

[38] Seo DO, Carillo MA, Chih-Hsiung Lim S, Tanaka KF, Drew MR (2015). Adult Hippocampal Neurogenesis Modulates Fear Learning through Associative and Nonassociative Mechanisms. J Neurosci.

[39] Meshi D (2006). Hippocampal neurogenesis is not required for behavioral effects of environmental enrichment. Nat Neurosci.

[40] Singer BH (2011). Compensatory network changes in the dentate gyrus restore long-term potentiation following ablation of neurogenesis in young-adult mice. Proc Natl Acad Sci USA.

[41] Zhang J, Jiao J (2015). Molecular Biomarkers for Embryonic and Adult Neural Stem Cell and Neurogenesis. Biomed Res Int.

[42] Furtado MB (2017). Mutations in the Katnb1 gene cause left-right asymmetry and heart defects. Dev Dyn.

[43] Sonbuchner TM, Rath U, Sharp DJ (2010). KL1 is a novel microtubule severing enzyme that regulates mitotic spindle architecture. Cell Cycle.

[44] Ververis A (2016). A novel family of katanin-like 2 protein isoforms (KATNAL2), interacting with nucleotide-binding proteins Nubp1 and Nubp2, are key regulators of different MT-based processes in mammalian cells. Cellular and molecular life sciences: CMLS.

[45] Lois C, Alvarez-Buylla A (1994). Long-distance neuronal migration in the adult mammalian brain. Science.

[46] Gage FH (2000). Mammalian neural stem cells. Science.

[47] Dayer AG, Ford AA, Cleaver KM, Yassaee M, Cameron HA (2003). Short-term and long-term survival of new neurons in the rat dentate gyrus. J Comp Neurol.

