# Supplementary material for: The Microtubule Severing Protein Katanin Regulates Proliferation of Neuronal Progenitors in Embryonic and Adult Neurogenesis
Source: Sci Rep. 2019 Nov 4;9:15940. doi: 10.1038/s41598-019-52367-3 (PMC6828949; doi:10.1038/s41598-019-52367-3)
Supplement: Supplementary file 1 — Supplementary Info [file 41598_2019_52367_MOESM1_ESM.pdf]

Supplementary Information

The Microtubule Severing Protein Katanin Regulates Proliferation of Neuronal Progenitors in Embryonic and Adult Neurogenesis

Franco Luis Lombino, Mary Muhia, Jeffrey Lopez-Rojas, Monika Brill, Edda Thies, Laura Ruschkies, David Lutz, Melanie Richter, Torben J. Hausrat, André T. Lopes, Francis J. McNally, Irm Hermans-Borgmeyer, Jessica E. M. Dunleavy, Sabine Hoffmeister-Ullerich[9], Michael Frotscher, Thomas Misgeld, Michael R. Kreutz, Froylan Calderon de Anda, Matthias Kneussel

Materials and Methods

Long range PCR. The correct insertion of the vector at the 3' and 5' arms of the targeted gene was tested using the following primers:

At the 5', primers a and b: 5'-CCAACTGACCTTGGGCAAGAACAT-3' and 5'-CAGCTGTGTTGCTGAAAATGAAGTATGGAA-3' led to an amplified band of 6226 bp.

At the 3', primers c and d: 5'-GAGATGGCGCAACGCAATTAAT-3' and 5'-TGACAAGCAGGGCTCTAAGTGGAG-3' led to an amplification product of 3762 bp.

Genotyping PCR. Extraction of genomic DNA was performed using Quickextract (Biozym) by incubation at 65°C and 95°C. DNA was then subjected to PCR using the following primers (Figure 1A; primers e and f):

94- 5'-CAAGATGGCTCATGCAGATAGATGTA-3'

170- 5'-ACTTTGGCTTCTGTTTATCTCCTTTCCT-3'.

236 bp (KO) and 270 bp (WT) amplified fragments were detected on a 4% agarose gel.

mRNA quantification. RNA extraction was performed using the Absolutely RNA Miniprep Kit (Agilent), with one cerebral cortex from E16 animals, each. Samples were homogenized by

shearing in 700 μl lysis buffer. Column-based purification including a DNase-treatment was performed according to the kit instructions. Total RNA was eluted in nuclease-free buffer and photometrically quantified by NanoDrop 1000 ND-1000 (Peqlab) measurement. Approximately 500-1000 ng RNA were analyzed on 1.2% Agarose gel with a 1-KB marker for overall RNA quality control. Reverse transcription was performed using 1μg total RNA, oligo dT primers and hexamer primers of the Superscript Reverse Transcription Kit (Life Technologies). qPCR reactions were performed with the Taqman gene expression master mix (Life Technologies). Primers were ordered from metabion international AG with purification grade desalted. qPCR reactions were performed on a 7900 HT Fast Real-Time PCR System (Applied Biosystems) in a 96-well format. qPCR reaction volumes were 20 μl. Thermal cycling conditions: activation of HotStarTaq DNA Polymerase at 95°C for 15 min and two-step cycling with denaturation at 94°C for 15 sec, annealing and extension at 60°C for 30 sec, and fluorescence data collection run in 40 cycles. After incubation at 95°C for 15 sec the melting analysis was performed from 60°C to 95°C with 2% ramp rate. Normalization of qPCR-based mRNA expression analysis was performed by using the Mm00441943_m1 taqman gene expression assay for the transferrin receptor (Life Technologies) as reference. Primer sequences: katexon11_for-5'-GGAATCTGCCTTAAGTCTTTGAAAA, reverse-5'-TCCAAATCACACCATTATGAAAGTTT; katexon11_probe-5'-FAM—AAATGTAGAA TTT(pdC)AGTGTA CGGAG---ZNA4—BHQ1. Differential gene expression was calculated with the REST© software according to the Pfaffl delta-delta Ct-Method[1].

Biochemistry and Western Blots. Cortices of E15 embryos and hippocampi of 2 months old animals were harvested and homogenized at 4°C in sucrose buffer (320 mM Sucrose, 1 mM $NaHCO_3$, 1 mM $MgCl_2$, 500 μM $CaCl_2$, 1 μM PMSF and Complete Roche protease inhibitor cocktail) with a potter, applying 14 strokes at 900 rpm. Brain homogenates were then centrifuged at 800 rpm for 10 min at 4°C. Supernatants were then quantified using BCA kit (Thermo, No. 23227) and concentrations were adjusted. Lysates were diluted in sample loading buffer (5X) 5% B-Mercaptoethanol, 0,02% Bromophenol Blue, 30% Glycerol, 10% SDS

and 250 mM Tris-HCl, pH 6,8) and boiled for 5-10 min at 95°C. Following SDS-gel electrophoresis, proteins were transferred into PVDF membranes, blocked in 5% milk and probed with different antibodies.

Histology, immunohistochemistry and microscopy. 2 months old males were euthanized with CO_2 and perfused using 4% PFA/PBS. Brains were harvested and post-fixed for 4-6 hours in 4% PFA/PBS. A 30% Sucrose/PBS solution was used for dehydration. Brains were then frozen embedded in Tissue Tek and 18 μm free floating sagittal sections were cut in the cryostat and conserved at -80°C until staining was performed. For immunostaining, sections were thawed for 20 min at room temperature and washed 3 times (always 5 min each in PBS). Permeabilization was performed by incubating 2 times for 10 min each in PBS / 0,5% Triton X-100. After a single washing step, unspecific epitopes were blocked for 1 hour in PBS containing 3% goat serum, 1% BSA and 0,05% Triton X-100. Primary antibody was incubated over night at 4°C diluted in Ab incubation buffer (PBS, 10% goat serum, 1% BSA, 0,05% Triton X-100). The following day, sections were washed three times and incubated for one hour with fluorescently tagged secondary antibodies in Ab incubation buffer. Eventually, antigen retrieval was performed (R&D, CTS013-4-5-6). *Nissl staining.* 18 μm sagittal sections were sequentially rinsed in 95%, 70% and 50% ethanol, washed twice in water and incubated for 2 min in Cresyl violet. Following a 1 min wash with water, additional incubations in 50% ethanol, 70% acid-ethanol (acetic acid), 95% ethanol and 100% ethanol were performed. Sections were then mounted with Entellan. *FluoroJadeC staining.* 18 μm sagittal sections were immersed in a basic alcohol solution (1% Sodium hydroxide, 80% ethanol) for 5 min and rinsed in 70% ethanol for 2 min. Tissue was washed for 2 min in ddH_2O and incubated in 0.06% potassium permanganate for 10 min. After, sections were washed again in water and incubated for 10 min in 0.0001% FluoroJade C solution dissolved in 0.1% acetic acid. After 3 washes with water, sections were air dried at 50°C, cleared in Xylene for 1 min and mounted with Aqua Poly Mount 1 (Polysciences). Images were taken with a confocal (Olympus FV-1000) or epifluorescent (Olympus SZX16) microscope, with equal settings across genotypes. Since cell numbers but

not fluorescence intensity was assessed, images have been brightness/contrast adjusted, if applicable. For Figure 3B, stacks of 36 slices of 0.5 µm each, were taken with a spinning disk confocal microscope with a 20x objective. Image deconvolution was performed for DCX and DAPI channels with Auto Quant X3 with an iteration of 10. Quantifications were performed with the Image J or MetaMorph software.

Sholl analysis. Sholl analysis was performed as described[2]. In brief, DiI-coated bullets were obtained by mixing 13.5 mg of DiI and 450 µl of methylene chloride. Solution containing DiI was mixed with gold particles (100 µl / 1 g) and methylene chloride was left to evaporate. Gold particles were then sonicated in the presence of 200 µl water for 10-30 minutes at room temperature in a water bath. TEZFEL tubes were pre-coated with 10 mg/ml polyvinylpyrolidone (PVP) and added with the gold particles by using a syringe. Mice were euthanized in the presence of CO_2 and perfused transcardially with 4% PFA / 0,5% Glutaraldehyde / PBS. Brain was harvested and hippocampus isolated. Subsequently, 300 µm sections were obtained with tissue chopper and post-fixed and maintained with 4% PFA / 0,5% Glutaraldehyde / PBS. Diolistic labeling was performed by shooting DiI-coated gold particles with Helios gene gun (BioRad) using 130 psi helium gas pressure. After labeling, overnight incubation in the dark was followed by imaging with 561-laser at the Spinning disk confocal microscope (Nikon, Visitron). Three-dimensional stacks were acquired with a step size of 2 µm. Occasionally, a binning of 2 was applied. Since cell morphology but not fluorescence intensity was assessed, images have been brightness/contrast adjusted, if applicable. For analysis, Fiji plugin Simple Neurite Tracer was used to map neuronal arborization. Virtual spheres at an interdistance of 10 µm was considered to analyze number of intersections.

Behavioral Experiments.

Elevated-plus maze test. The arena was made of PVC and was formed by 4 arms positioned at 78 cm from the floor. Each arm was 30 cm long and 5 cm width. The center of the arena was a 5 cm^2 square. Two of the arms were completely open (open arms) and the other two,

surrounded by opaque walls 16 cm high (closed arms). During the experiment, the animal was placed in the center of the arena facing one of the closed arms and video-recorded by a digital camera positioned above. The arena was illuminated by lamps that provided 50 Lux of even light. Each trial lasted 5 min and video tracking was performed using the Ethovision (Version XT 8.5, Noldus Technology; The Netherlands) software for windows. The percentage of time in open arms was calculated as= time in open arms/time in all arms x 100.

Light-dark transition test. A 45 cm x 20 cm x 20 cm opaque PVC box was divided into a light and a dark compartment. The dark compartment was achieved by adding a smaller box of 19,5 cm x 19,5 cm x 15 cm on one extremity of the arena. The dark box had an open side facing the light compartment of 7 cm x 7 cm in order to allow the mouse transition from one space to the other. Lamps providing 300 Lux were placed above the arena. During testing, the animal was placed in the dark compartment from the top and was free to transit from one area to the other. 10 min trials were video-recorded by a digital camera placed above. Ethovision for windows allowed video tracking. The time spent in the lit chamber was considered as a measurement of anxiety-related behavior[3].

Open-field test. A box made of PVC containing 4 arenas of 50 cm x 50 cm x 40 cm each, was illuminated by 4 lamps which provided 50 Lux evenly in all the arenas. A digital camera was placed above and used for video recording of the whole session. Each arena was virtually divided into a central region, a medial region and an outer region. Each experimental trial lasted 40 minutes and 2 males and 2 females were tested simultaneously. Video tracking was performed using Ethovision for windows. Distance moved in cm was an indication of correct locomotor activity and was binned to 5 min time points. Percentage of time in the center zone was calculated as time in center zone / total time x 100.

Spontaneous alternation test. The Y-maze apparatus consisted of three identical arms (length × width × height: 37.5 cm x 7.5 cm x 16 cm) made of opaque beige Plexiglas and interconnected at 120 degrees from a central triangle. Mice were placed in one arm of the maze and allowed to explore freely for 6 min during which the number and sequence of arm entries was noted down. Total number of arm entries was used as a measure for activity. The

number of alternations, defined as successive entries into each of the three arms as an overlapping triplet set was calculated. Percentage alternation scores were then computed as follows: Spontaneous alternation (%) = [(Number of alternations)/ (Total number of arm entries-2)] x 100.

Statistical Analyses

All animals used for the experiments were either katanin p60 +/+ and +/-, and katanin p80 +/+ and -/-. At least three age-matched animals per genotype were analyzed. For mRNA quantification and in-utero electroporation, the sex of the animals was not determined. For electrophysiology and behavior, males and females age-matched were considered. In details, LTP measurements were performed in a group or 4 females (+/+=2; +/-=2) and 4 males (+/+=2; +/-=2) between 2 and 3 months of age. Behavioral experiments were performed in two different cohorts between 2 and three months of age. Cohort 1 was composed of 11 males (+/-=5; +/+=6) and 11 females (+/-=5; +/+=6) and was used for elevated-plus maze, light-dark transition test and open field. Cohort 2 included 18 males (+/-=10; +/+=8) and 10 females (+/-=5; +/+=5). mRNA levels were analyzed statistically with the REST© software according to the Pfaffl delta-delta Ct-Method. SPSS software for Windows (version 13.0, SPSS Inc. Chicago, IL, USA) was used to perform statistical analyses of behavioral experiments. We performed analysis of variance (ANOVA) with the factor between-subject genotype (+/+) versus (+/-), and whenever appropriate bins of trials or days were included as repeated measurements. Western blots, inmunohistochemistry, in-utero electroporation and Sholl's analysis were analyzed with either parametric or non-parametric T-tests as indicated in figure legends. Data was represented as ± Standard Error of Means (S.E.M.). A p-value of <0.05 was considered significant and was represented in the figures as *, and so $p<0.01$ ** and $p<0.001$ ***.

References

1 Pfaffl, M. W. A new mathematical model for relative quantification in real-time RT-PCR. *Nucleic Acids Res* **29**, e45 (2001).

2 Muhia, M. *et al.* The Kinesin KIF21B Regulates Microtubule Dynamics and Is Essential for Neuronal Morphology, Synapse Function, and Learning and Memory. *Cell Rep* **15**, 968-977, doi:10.1016/j.celrep.2016.03.086 (2016).

3 Crawley, J. & Goodwin, F. K. Preliminary report of a simple animal behavior model for the anxiolytic effects of benzodiazepines. *Pharmacol Biochem Behav* **13**, 167-170 (1980).

Supplementary Figure 1

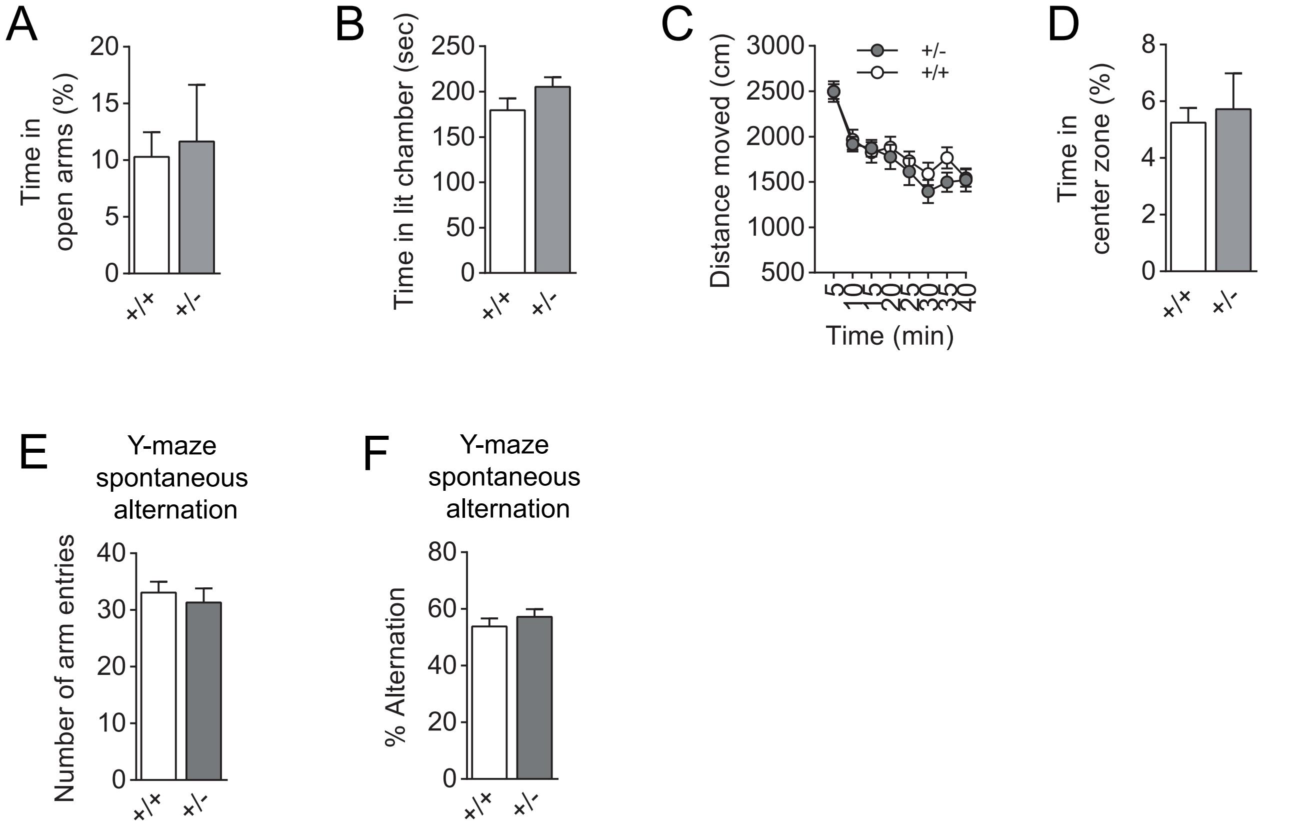

Supplementary Figure 1. Katanin p60 haploinsufficiency does not affect spontaneous alternation, spatial place discrimination or contextual fear conditioning.
(A) Assessment of anxiety-like behavior in the elevated plus-maze test yielded comparable percentage time spent in the open aversive arms between +/+ and +/- mice. (B) Similarly, time spent in the brightly lit chamber of the light-dark transition test was comparable for both genotype groups. (C) Measures for locomotor activity and habituation as a function of distance travelled across a 40 min open-field test session is comparable for +/+ and +/- mice. (D) The proportion of time spent in the central zone of the open-field arena as an index for anxiety-like behavior was similar for +/+ and +/- mice. (E) Comparable total arm entries in the Y-maze spontaneous alternation task. (F) Working memory function as indexed by percentage alternation scores in heterozygous (+/-) mice matches control (+/+) mice.

Supplementary Figure 2

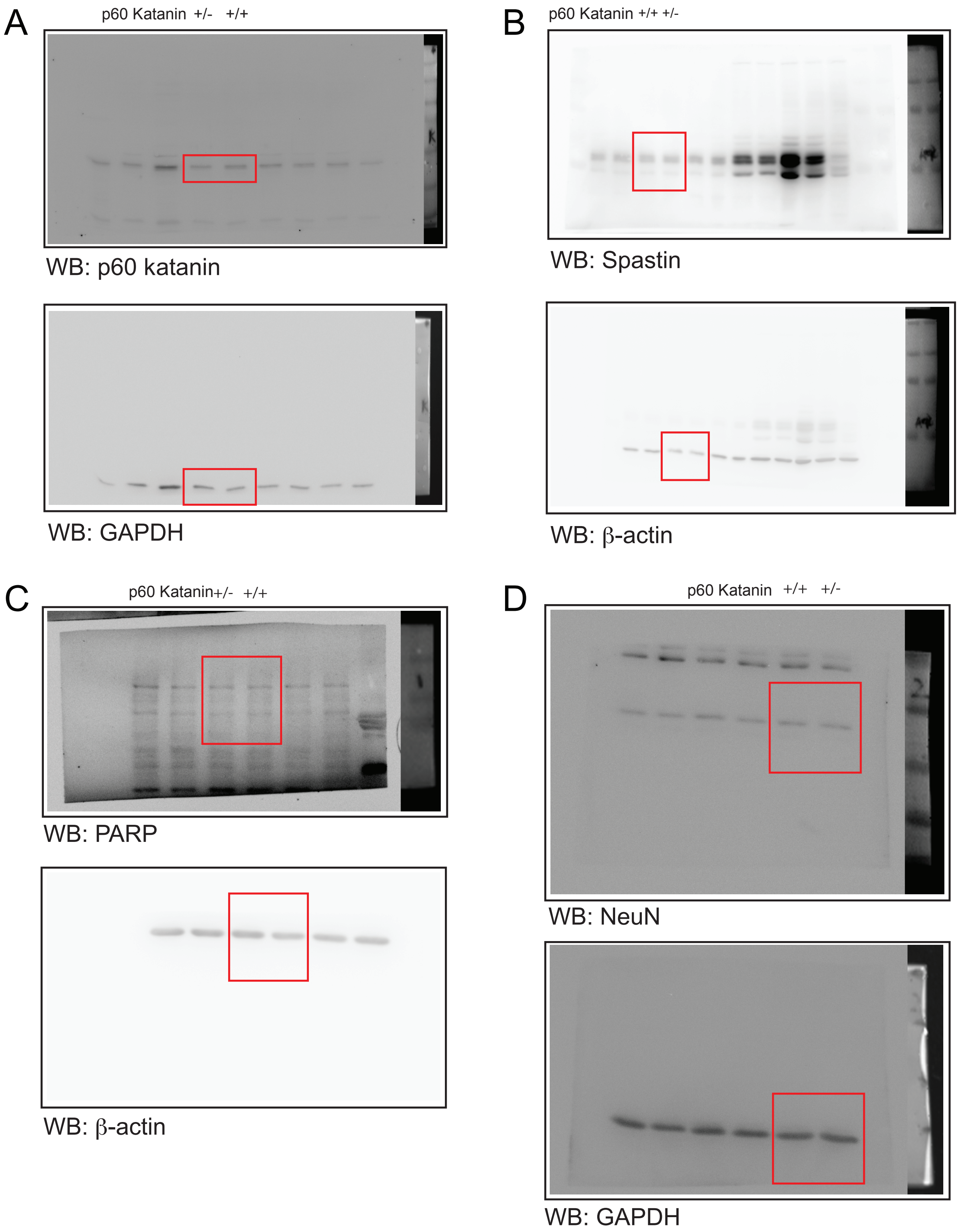

Supplementary Figure 2. Area used for representative figure shown in red square. (A) Full membrane scans for representative figures 1E and F (for p60 katanin and GAPDH, levels were adjusted due to weak signal). (B) Full membrane scans for figure 1I and J. (C) Full scans for figure 1M and N (for PARP, levels were adjusted due to weak signal). Membrane was cut prior to antibody incubation. (D) Full scans for figure 3E and F (for NeuN and GAPDH, levels were adjusted due to weak signal).
